# Dental Luting Cements and Peri-Implantitis: Molecular Mechanisms, Clinical Implications and the Role of Autophagy and Nanotechnology—A Narrative Review

**DOI:** 10.3390/dj14080485

**Published:** 2026-08-05

**Authors:** Adriana Bucată, Lucian Toma Ciocan, Alexandra Ripszky, Mihaela Tănase, Rădulescu Radu, Melis Izet, Ana Cernega, Marina Meleşcanu Imre

**Affiliations:** 1 Pedodontics Department, Faculty of Dental Medicine, “Carol Davila” University of Medicine and Pharmacy, 041292 Bucharest, Romania; adriana.bucata@drd.umfcd.ro (A.B.); mihaela.tanase@umfcd.ro (M.T.); 2 Discipline of Dental Prosthesis Technology, Faculty of Dentistry, “Carol Davila” University of Medicine and Pharmacy, Dionisie Lupu Street, No. 37, District 2, 020021 Bucharest, Romania; lucian.ciocan@umfcd.ro; 3 Department of Biochemistry, Faculty of Dental Medicine, “Carol Davila” University of Medicine and Pharmacy, 37 Dionisie Lupu Street, District 2, 020021 Bucharest, Romania; 4 The Interdisciplinary Center for Dental Research and Development, Faculty of Dental Medicine, “Carol Davila” University of Medicine and Pharmacy, 19-21 Jean Louis Calderon Street, 020021 Bucharest, Romania; melis.izet@umfcd.ro; 5 Department of Professional Organization and Medical Legislation-Malpractice, “Carol Davila” University of Medicine and Pharmacy, 020021 Bucharest, Romania; ana.cernega@umfcd.ro; 6 Department of Complete Denture, Faculty of Dental Medicine, “Carol Davila” University of Medicine and Pharmacy, 17-23 Plevnei Street, 010221 Bucharest, Romania; marina.imre@umfcd.ro

**Keywords:** cemented crown vs. screw-retained crowns, dental luting cement, peri-implantitis, autophagy, nanotechnology, nanomaterials

## Abstract

**Background and Objectives**: This narrative review aims to evaluate the correlation between dental luting cements, peri-implantitis and the molecular mechanisms governing tissue destruction, with a specific focus on the role of autophagy and nanotechnology. **Methods**: A comprehensive literature search was conducted across electronic databases, including PubMed, Scopus and Web of Science, to identify relevant studies on cement cytotoxicity, cellular responses and nanomaterial integration. **Results**: Residual cement in the peri-implant sulcus promotes biofilm accumulation and severe inflammation. At a cellular level, resin-base monomers (e.g., Bis-GMA) impair human gingival fibroblasts via oxidative stress and mitochondrial dysfunction. Autophagy serves as a vital cytoprotective mechanism against cement toxicity and titanium particle accumulation. Experimental studies suggest that incorporating nanomaterials, specifically graphene oxide and silver nanoparticles (≤1 wt.%), has shown promising results in enhancing antimicrobial efficacy without compromising biocompatibility in vitro. **Conclusions**: To minimize peri-implantitis, clinical protocols should prioritize the meticulous removal of excess cement and the development of nano-reinforced luting agents. Optimizing these molecular pathways offers actionable preventive strategies, guiding clinicians toward safer cementation protocols and enhanced long-term implant success.

## 1. Introduction

Implant-supported restorations have become a predictable and widely used solution for rehabilitating edentulous spaces, with long-term survival rates frequently reported above 90% [1]. Nowadays, there are two types of retention for the implant restorations, cement-retained and screw-retained; the choice of the optimal system remains a subject of debate [2].

The cement-retained restorations offer important clinical advantages such as superior esthetics and easier passive fit compared with screw-retained restorations. The disadvantages include difficulty of removing permanently cemented restorations, retention of excess cement, difficulty of subgingival control, and increased risk of peri-implant inflammation. The biological risks associated with the use of cements remain insufficiently controlled [3,4].

On the other hand, the screw-retained restorations (TDPs) have major advantages such as reducing the risk of cement-induced peri-implantitis and efficient distribution of occlusal forces, with a lower risk of mechanical complication [5]. Angled screw-retained crowns (ASCs) offer similar implantation and esthetic results to cemented crowns, with the possibility of deviating the access channel up to ~25 degrees to avoid a buccal emergence in the esthetic zone. The clear biological advantages are reduced bleeding on probing and the ability to recover the prosthetic restoration [4].

Therefore, the biological success of implant restorations is not determined only by osseointegration but also by selecting the correct retention system [2] and maintaining peri-implant tissue health. In this context, peri-implantitis remains one of the most important biological complications, characterized by chronic inflammation and progressive marginal bone loss, which may eventually lead to implant loss [6,7,8,9,10].

Initially, under the influence of biofilm, the inflammation of the mucosa surrounding the implant occurs; subsequently, due to the persistence of the inflammatory infiltrate, the peri-implant osteitis ensues [11]. Also, the etiology of peri-implantitis is multifactorial, involving complex interactions between bacterial biofilm, the host immune response, mechanical factors and the characteristics of biomaterials used. Over the last decade, residual dental cement has been recognized as a significant risk factor, especially in cemented restorations where detection and complete removal of subgingival cement can be difficult [3,12,13,14,15]. Clinical and histopathological studies have shown that residual cement particles can act as a substrate for biofilm accumulation and as a persistent stimulus for the inflammatory response [6,16,17,18,19,20,21,22,23,24]. Cement-induced peri-implantitis is responsible for approximately 1.9–75% of dental implant failures linked to peri-implant diseases [25]. Crucial to this context is the distinction between peri-implant mucositis, a reversible inflammatory lesion confined to the soft tissue, and peri-implantitis, which involves progressive, irreversible marginal bone loss [14]. This broad range stems from the high heterogeneity in clinical study designs, notably differing follow-up periods (with early recalls capturing initial peri-implant mucositis vs. long-term retrospective studies tracking severe bone loss), variations in diagnostic criteria and the distinct chemical and radiographic properties of the dental cements evaluated [25].

In this review, to ensure precise scientific communication, it is necessary to establish three terms often used interchangeably, based on the clinical and chronological stages of cement extrusion:

- “Cement excess” refers specifically to the initial volume of the luting agent that overflows from the crown margin immediately upon seating the restoration.
- “Remnant cement” denotes the small clinical fragments or debris that inadvertently escape the clinician’s initial cleaning process in the dental office.
- “Residual cement” describes the long-term, structurally undetected cement that remains subgingivally over time, acting as a persistent stimulus for foreign body reaction and peri-implantitis.

The main categories of luting cements used in implant restorations include zinc oxide eugenol (ZOE) cements, non-eugenol zinc oxide (ZOE NE) cements, zinc phosphate cements (ZP), glass ionomer cements (GIC), resin-modified glass ionomer cements (RMGIC), zinc polycarboxylate (ZPC) and resin luting cements and self-adhesive resin cements (RCs) [3]. The luting materials differ in retention [26,27], solubility [28,29], biocompatibility and difficulty of excess removal [9,30,31].

For example, ZOE cement is biocompatible, has antibacterial effect, is soluble and radiopaque—ideal for provisional restorations—but has very low retention and may leave a gap at the prosthetic abutment–crown interface. ZOE NE is hypoallergenic and does not interfere with subsequent resin cement polymerization, but shows greater microleakage than ZOE, with high risk of retention loss [3].

ZP cement, considered semi-permanent, does not chemically bond to titanium, has poor marginal seal, is soluble initially, but is highly radiopaque, has good retention, and its excess can be removed easily. Glass ionomer cements have moderate retention, are moisture-sensitive during setting, and are contraindicated in areas with high occlusal forces and dimensional instability. RMGICs, in the permanent cement category, have good retention and radiopacity, but high water uptake and subsequent removal of excess can be difficult [3,28]. ZP cements pose a higher biological risk due to microleakage and corrosive behavior in contact with titanium. Resin cements present high retention and insolubility, but excess is difficult to remove and they have the highest incidence of peri-implantitis [3]. Material composition, polymerization degree, solubility and radiopacity all influence molecular outcomes in peri-implant tissues [32]. Table 1 shows all those properties and moreover the advantages and disadvantages of various luting cement agents [3,6,7,22,28,33,34,35].

### 1.1. Aims of the Review

While several comprehensive reviews in the literature, such as that by Heboyan et al. [33], have extensively mapped the general classification, biomechanical properties, handling protocols and adhesive strengths of dental luting cements on natural teeth, there remains a critical gap in correlating these materials specifically with peri-implant pathology. Unlike previous overviews, the present narrative review focuses on the biological and molecular repercussions of residual cements in the peri-implant sulcus. More precisely, a link is forged between clinical prosthetic complications (peri-implantitis) and cellular response, highlighting the dual role of autophagy as a cytoprotective mechanism against monomer-induced cytotoxicity and the potential implementation of nanotechnology (such as graphene-base biomaterials) suggested by preclinical evidence to optimize cement biocompatibility and antimicrobial efficacy.

Therefore, the primary aim of this narrative review is to critically evaluate the molecular and cellular mechanisms underlying residual luting cement-induced peri-implantitis. Furthermore, the scope of this study extends to examining the dual potential of modulating autophagy pathways and incorporating advanced nanotechnology to mitigate cement cytotoxicity, enhance biocompatibility and improve clinical outcomes in implant dentistry.

### 1.2. Search Strategy and Selection Criteria

This narrative review is based on a comprehensive and reproductible literature search spanning the following three major electronic databases: PubMed, Web of Science and Scopus. Conducted between 10 December 2025 and 20 April 2026, the baseline search criteria restricted results to open-access articles published in English within the 2016—2026 timeframe. However, to ensure internal consistency and comprehensive theoretical coverage, the landmark foundational literature from 2015 was exceptionally integrated into the review.

The exact search strings utilized in each database consisted of Medical Subject Headings (MeSHs) terms and specific keywords combined with Boolean operators (“AND”/”OR”) as follows:

- PubMed: (“dental cements” [MeSH Terms] OR “luting cements” [All Fields]) AND (“peri-implantitis” [MeSH Terms] OR “peri-implant disease” [All Fields]) AND (“autophagy” [MeSH Terms] OR “nanotechnology” [MeSH Terms] OR “nanomaterials” [All Fields]);
- Web of Science/Scopus: TS = ((“luting cement*” OR “dental cement*”) AND (“peri-implantitis” OR “cement-retained restorations”) AND (“autophagy” OR “nanotechnology” OR “nanoparticles” OR “graphene oxide”)).

To be included in this critical analysis, studies had to meet the following criteria: (1) original experimental in vitro or in vivo articles, clinical trials and prospective/retrospective cohort studies; (2) target population focused on implant-supported restorations and peri-implant tissues; (3) studies investigating the molecular, cytotoxic, mechanical or biological effects of residual luting cements and (4) studies evaluating the role of autophagy or nanotechnology in mitigating cement toxicity. Exclusion criteria were: (1) studies focusing exclusively on cementation protocols for natural teeth without relevance to dental implants; (2) articles published in languages other than English; (3) duplicate publications and (4) non-peer-reviewed white paper or conference abstracts with insufficient data.

**Figure 1:**
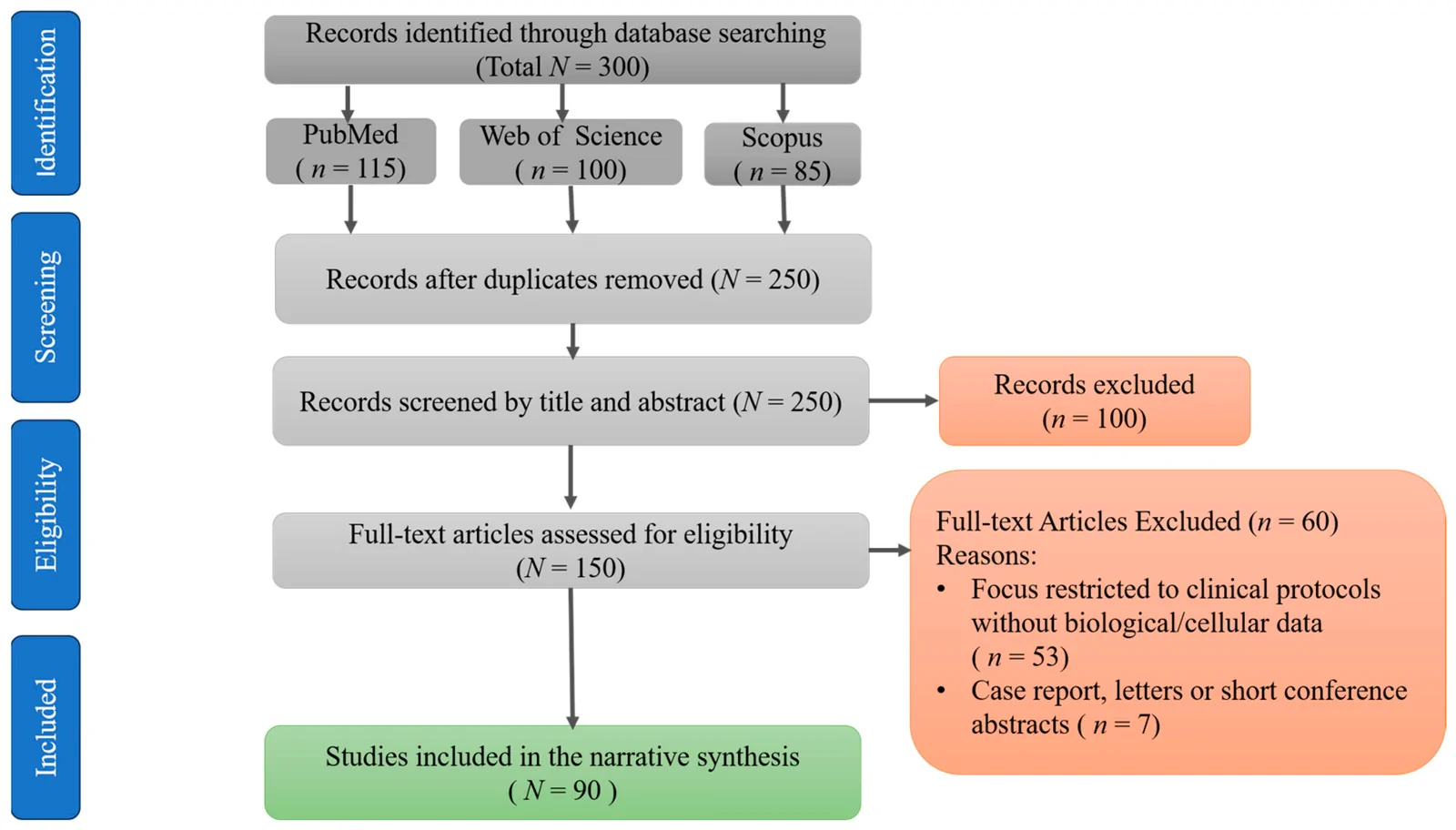
PRISMA-style flow diagram. Note: *N* = total number of articles; *n* = subgroup of articles.

The selection process followed a structured screening cascade. A total of 300 records were initially identified across the three databases (PubMed: 115, Web of Science: 100, and Scopus: 85). After the exclusion of 50 duplicated entries, a total of 250 articles underwent independent title and abstract screening by two researchers. Of these, 100 were excluded for not aligning with the core scope of this review. The remaining 150 full-text articles were comprehensively evaluated and 60 were excluded due to a lack of focus on biological/molecular pathways or peri-implantitis outcomes. Ultimately, 90 articles were deemed highly relevant and included in the narrative synthesis. The complete study selection process and the detailed screening cascade are visually illustrated in Figure 1.

## 2. Peri-Implant Tissue Homeostasis: A Molecular Perspective

The molecular mechanisms by which luting materials influence peri-implant tissue homeostasis are not fully elucidated [6,9,28,34,35,36]. The scientific literature describes two pathways, direct and indirect, through which excess cement is involved in the etiopathogenesis of peri-implant disease. In the direct pathway, both chemical and mechanical mechanisms may be involved. Given the varying biocompatibility of these materials, cement may act as an allergenic agent, initially inducing sensitization and subsequently provoking a localized reaction [37]. Beyond direct cytotoxicity, luting materials indirectly alter peri-implant homeostasis by favoring pathogenic biofilm formation [37,38].

Peri-implant tissue homeostasis depends on a fragile equilibrium between epithelial barrier integrity, connective tissue remodeling, immune surveillance and bone metabolism [39]. Unlike periodontal tissues, peri-implant mucosa lacks Sharpey fiber insertion and has fewer blood vessels, rendering it more vulnerable to chemical and inflammatory insults [40]. Molecularly, this balance is governed by epithelial junction proteins (integrins, laminins), fibroblast-derived extracellular matrix (ECM) proteins, cytokine gradients and osteo-immune signaling pathways that regulate bone turnover [39,40].

### 2.1. Cytotoxic Effects on Peri-Implant Soft-Tissue Cells

Luting materials, especially when incompletely removed, can disrupt this equilibrium by acting as chemical stressors, inflammatory triggers, and biofilm-retentive substrates, initiating molecular cascades that impair tissue stability.

At the cellular level, human gingival fibroblasts (HGFs) and epithelial cells (ECs) are the first responders to cement exposure. Multiple in vitro studies show that commonly used luting materials release zinc ions, methacrylate monomers, eugenol derivatives and acidic by-products, which reduce cell viability and alter cellular metabolism [19,41].

Mechanistically, resin and resin-modified cements have been shown to induce mitochondrial dysfunction, increased Reactive Oxygen Species (ROS) and apoptosis via caspase activation [22].

Bajantri et al. investigated the biological effects of four resin cements used in implant prosthetics on HGF. SoloCem, a chemically setting resin cement, showed the highest cytotoxicity—reducing fibroblast viability and migration and rapidly increasing ROS—whereas Multilink Hybrid Abutment (self-cure) showed the best biocompatibility with minimal effects. Two dual-cure cements tested induced moderate cytotoxicity and oxidative stress at high concentrations as follows: RelyX Unicem 2 showed more pronounced effects, while Panavia V5 affected viability early in exposure in a dilution-dependent manner [19].

A study evaluating residual monomer release from these three self-adhesive resin cements (G-Cem, GC Corp., Tokyo, Japan; SpeedCem, Ivoclar Vivadent, Schaan, Liechtenstein; RelyX U200, 3M ESPE, St. Paul, MN, USA) found that all materials triggered programmed cell death processes through ROS generation, with RelyX U200 having the greatest potential to affect DNA integrity [42].

An editorial in a specialized journal noted that provisional eugenol-free cements have more favorable soft-tissue responses if completely removed [32]. Furthermore, an in vitro study tested the cytotoxicity of 12 resin cements compared to conventional ZP cement on human cell lines relevant to dentistry and implantology (oral epithelial cells, endothelial cells, periodontal ligament fibroblasts and two osteoblastic cell lines). Osteoblastic cells were most affected, showing the greatest viability decrease, indicating heightened sensitivity of bone tissue to monomers released from resin-based cements (e.g., Hydroxyethyl methacrylate (HEMA), Triethylene glycol dimethacrylate (TEGDMA), Bisphenol A-glycidyl methacrylate (bis-GMA), and Urethane dimethacrylate (UDMA)). For this reason, the author emphasizes cautious use of these cements, correct polymerization, and complete removal of excess cement; zinc phosphate cements appear better tolerated in vitro biologically [20]. Another study, investigating the chemical composition of resin-based cements, has demonstrated that Bis-GMA exhibits the highest cytotoxic potential toward human and murine fibroblasts when compared with other commonly used monomers such as UDMA, TEGDMA and HEMA. The authors explain this heightened cytotoxicity through several distinct chemical and molecular pathways. From a structural standpoint, Bis-GMA possesses a rigid, hydrophobic bisphenol A (BPA) core and a high lipophilicity, which facilitates its rapid penetration through the lipid bilayer of the cell membrane, altering membrane permeability and integrity. Once intracellular, Bis-GMA significantly induces production of ROS, leading to severe mitochondrial dysfunction and subsequent activation of the caspase-dependent apoptotic cascade. Furthermore, due to its molecular weight and chemical structure, unpolymerized Bis-GMA monomers show a higher tendency to deplete intracellular glutathione (GSH) levels compared to TEGMA or HEMA, drastically impairing the cell’s antioxidant defense mechanism and leading to persistent oxidative DNA damage and cell cycle arrest [43].

Bajoria et al. [20] also demonstrated a direct relationship between cement excess and defects in bone mineralization. The authors state that nano-integrated bio-ceramic cement (NIB) is the most biocompatible and has the least negative impact on bone mineralization, making it suitable for cementing restorations on implants, whereas GIC and ZP cements show moderate toxicity and should be avoided in such cases.

Additionally, the literature reports that ZOE cements exhibit strong cytotoxicity through membrane destabilization and inhibition of fibroblast proliferation, despite high antibacterial benefits. Gingival fibroblasts are significantly more susceptible than osteoblasts, indicating early soft-tissue breakdown as a key pathogenic event [19,41,44]. Loss of fibroblast viability compromises collagen synthesis (types I and III), weakening the peri-implant connective tissue seal. Epithelial attachment to implant abutment relies on hemidesmosomes, integrins (α6β4) and laminin-332-mediated adhesion. Toxic components eluted from luting materials downregulate these adhesion molecules while promoting epithelial cell migration and ulceration [40].

**Figure 2:**
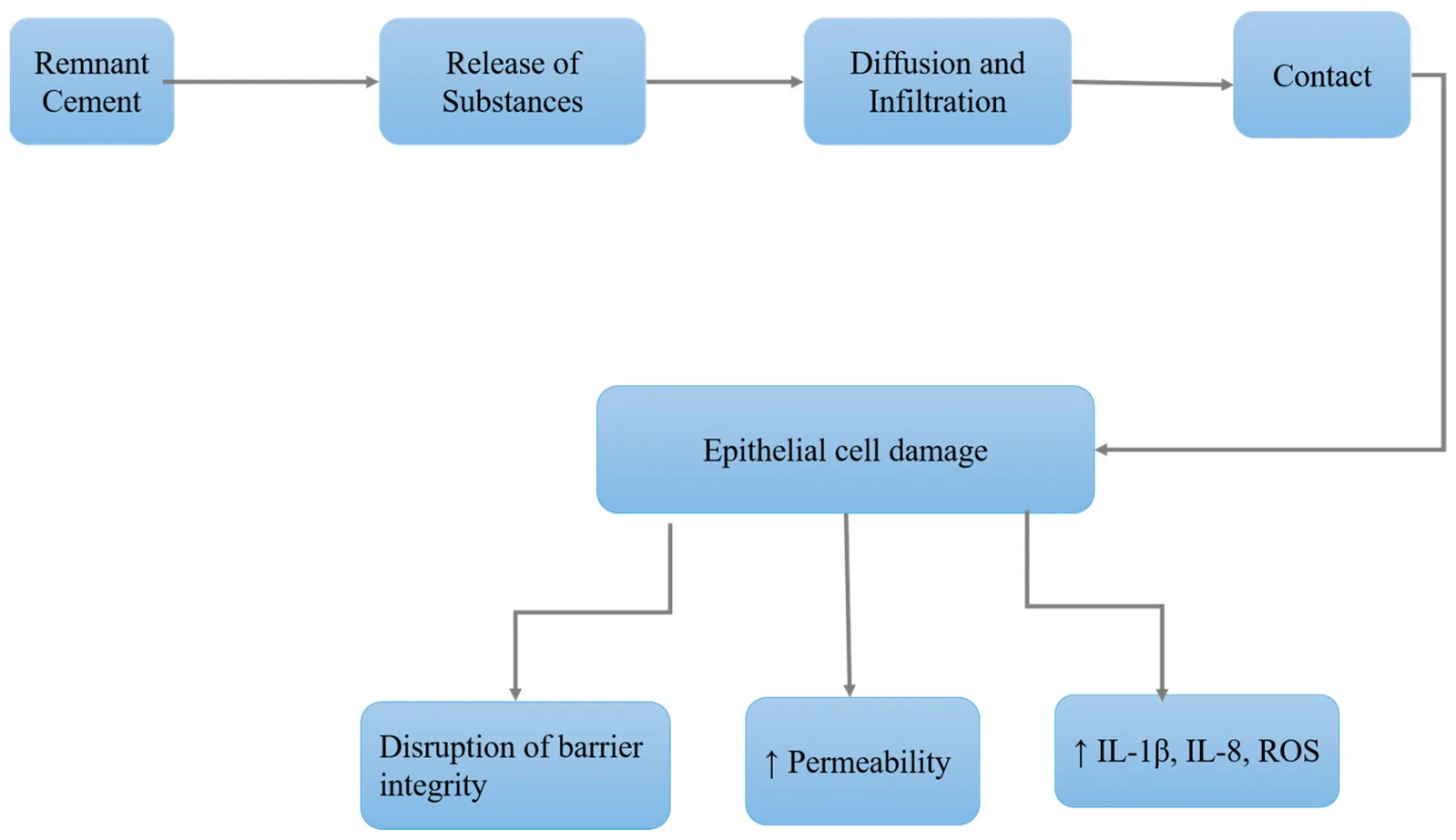
Mechanistic diagram of the biological pathways involved in cement-induced peri-implantitis. Note: The upper arrows (↑) denote an increase/elevation in the respective biological parameter. Abbreviations: IL 1β/6/8—interleukin 1β/6/8; ROS—Reactive Oxygen Species.

The cytotoxic effects of dental cements on gingival fibroblasts and epithelial cells in implant-supported restorations are illustrated in the figure below (Figure 2).

### 2.2. Chemical and Electrochemical Corrosion Pathways

Beyond the microenvironmental and microbiological shifts, the progression of peri-implantitis is driven by distinct chemical and electrochemical corrosion pathways that compromise the structural integrity of the implant. In the complex oral cavity, the thin, passive titanium dioxide (TiO_2_) film undergoes thermodynamic instability and local breakdown under specific chemical conditions.

Microbial cell density and anaerobic multi-species agglomerates produce organic metabolites, primarily lactic and formic acids, which directly acidify the peri-implant microenvironment, dropping the localized pH values to levels as low as 4.0 or even 2.0. In these highly acidic regions, the protective TiO_2_ passive film undergoes chemical alteration and localized dissolution, leading to pitting and crevice corrosion, particularly within restricted prosthetic connection margins where oxygen depletion is prevalent. Additionally, the presence of fluorides (originating from ordinary fluoridated toothpastes or prophylactic gels) chemically interacts with the compromised oxide film. When high F^−^ concentrations combine with an acidic medium, they synthesize hydrofluoric acid (HF), a highly reactive chemical agent that aggressively attacks and destroys the titanium passive layer [45]. Furthermore, hydrogen peroxide (H_2_O_2_), produced both by bacteria and host leukocytes during active peri-implant inflammatory responses, acts as a strong chemical oxidizer that alters the oxide layer capacitance and increases ion exchange with the electrolyte medium.

Once the passive film is disrupted, the continuous chemical attack suppresses stable self-repair, converting the oxide into an active film. This progressive chemical and electrochemical dissolution yields hydrated titanium oxides (such as Ti(OH)_3_^+^) and causes the sustained release of metallic ions (Ti^4+^) along with micro- and nanoparticles into the surrounding soft tissues. At the biological interface, the accumulation of this metallic debris serves as a chronic chemical irritant that stimulates inflammatory cells, including macrophages and neutrophils. This interaction accelerates the formation and activation of osteoclasts and alveolar bone resorption, completing the synergistic chemical–biological cascade that defines peri-implantitis [46].

### 2.3. Mechanical Consequences of Residual Cement

Moreover, residual cement in the sulcus causes chronic mechanical irritation, which perpetuates epithelial permeability and allowing bacterial components to penetrate deeper tissues [30]. This results in the sustained activation of toll-like receptors (especially TLR-4) [47], thereby escalating inflammatory signaling even in the absence of overt plaque accumulation [25].

**Figure 3:**
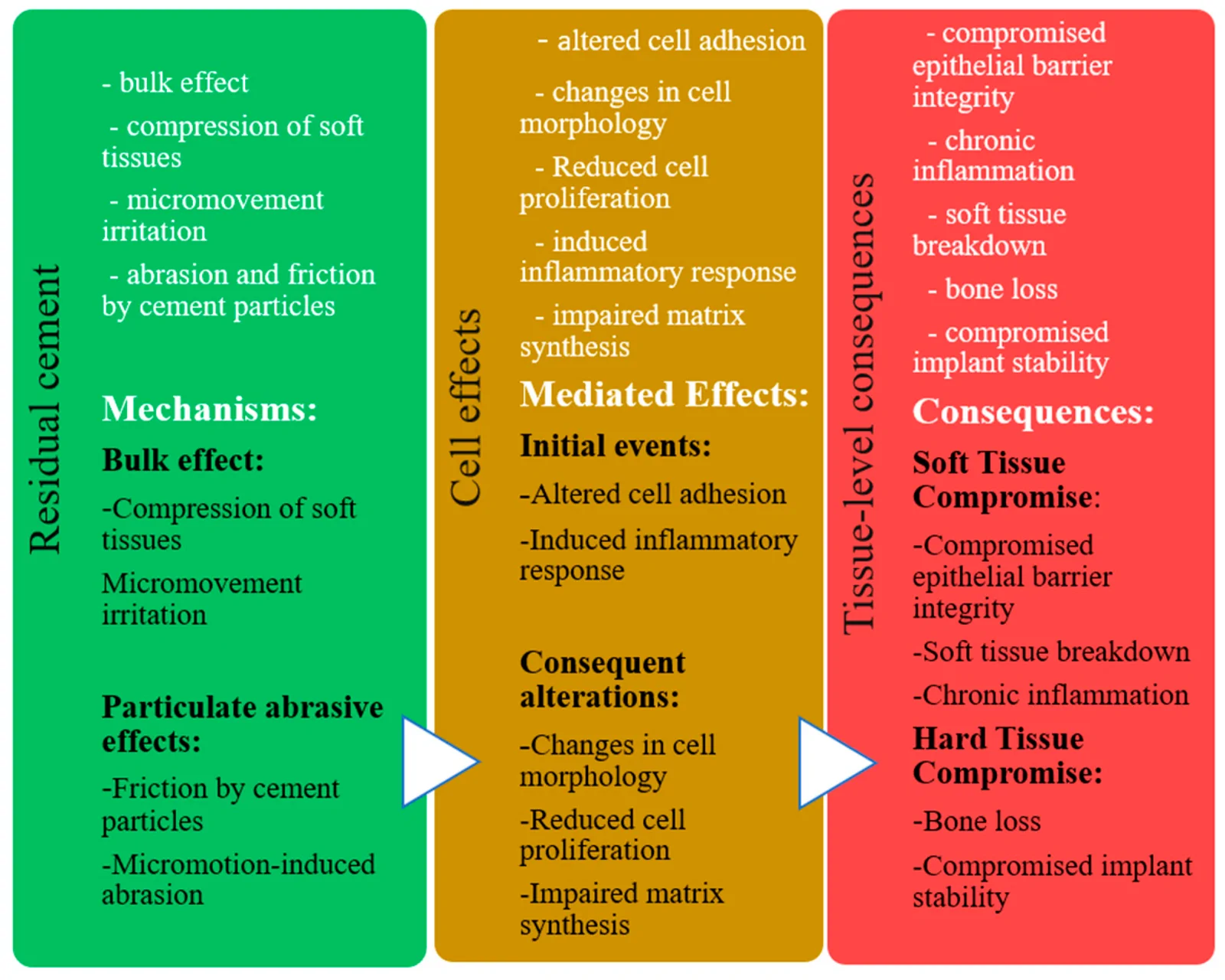
The diagram summarizes the mechanical effects of residual cement on peri-implant cells and tissues. Residual cement induces mechanical irritation and compression, leading to cellular dysfunction, inflammatory responses, tissue breakdown, bone loss and impaired implant stability.

Luting materials influence peri-implant homeostasis by reshaping the local immune microenvironment. Cement remnants serve as persistent foreign bodies, leading to the continuous recruitment of macrophage and neutrophil [38,48] (Figure 3).

Animal and histopathological studies demonstrate that cement particles surrounded by an inflammatory infiltrate are associated with localized osteolysis and reduced bone-to-implant contact, confirming a direct link between luting materials and peri-implant bone loss [25,49]. Furthermore, surface roughness variations may also influence cellular viability; specifically, lower roughness values ranging between 0.2 and 0.8 μm have been associated with reduced cytotoxic effects from resin-based cements [43].

Several distinct molecular pathways are involved in peri-implantitis caused by luting agents [23]. The primary mechanism involves gingival inflammation triggered by macrophage activation which prompts the release of key pro-inflammatory mediators, such as interleukin-1β (IL–1β), tumor necrosis factor–α (TNF–α), interleukin-6 (IL–6), that stimulate osteoclasts and drive bone resorption [47].

These molecular events are furthermore amplified by the nuclear factor kappa (NF–kB) pathway, which elevates the secretion of matrix metalloproteinases (MMP-8, MMP-9) by fibroblasts and macrophages, accelerating connective tissue degradation [48,50]. Concurrently, the decreased expression of anti-inflammatory mediators, including interleukin–10 (IL–10) and Transforming Growth Factor–β (TGF–β), may impair the resolution of inflammation [38].

Oxidative stress serves as an additional pathogenic pathway: the rough surfaces of the residual cement and biofilm can generate excessive ROS, causing DNA damage, cellular dysfunction and chronic inflammation [15,51]. While ROS is essential for maintaining normal cellular functions, its primary intracellular sources under stress conditions remain NADPH oxidase (NOX), mitochondria, peroxidase and endoplasmic reticulum [52].

### 2.4. Effects of Cement Excess and Associated Biofilm on Peri-Implantitis

Residual cements create micro-retentive niches that protect anaerobic bacteria [30] and provide surfaces highly conductive to the adsorption of salivary proteins and bacterial adhesins [53]. The microorganisms involved are often Gram-negative anaerobes, such as *Porphyromonas gingivalis* (*PG*), *Tannerella forsythia*, *Treponema denticola*, *Fusobacterium nucleatum* and *Prevotella intermedia* [53,54].

Dysbiotic biofilms release lipopolysaccharides (LPSs), which amplify inflammatory signaling through TLR-mediated pathways. This LPS-induced cytokine release synergizes with cement-induced inflammation, intensifying tissue destruction [38]. Clinically, an excess of cement is associated with pronounced gingival inflammation characterized by bleeding on probing, radiographic evidence of bone loss and implant mobility in advanced stages [23,53].

**Figure 4:**
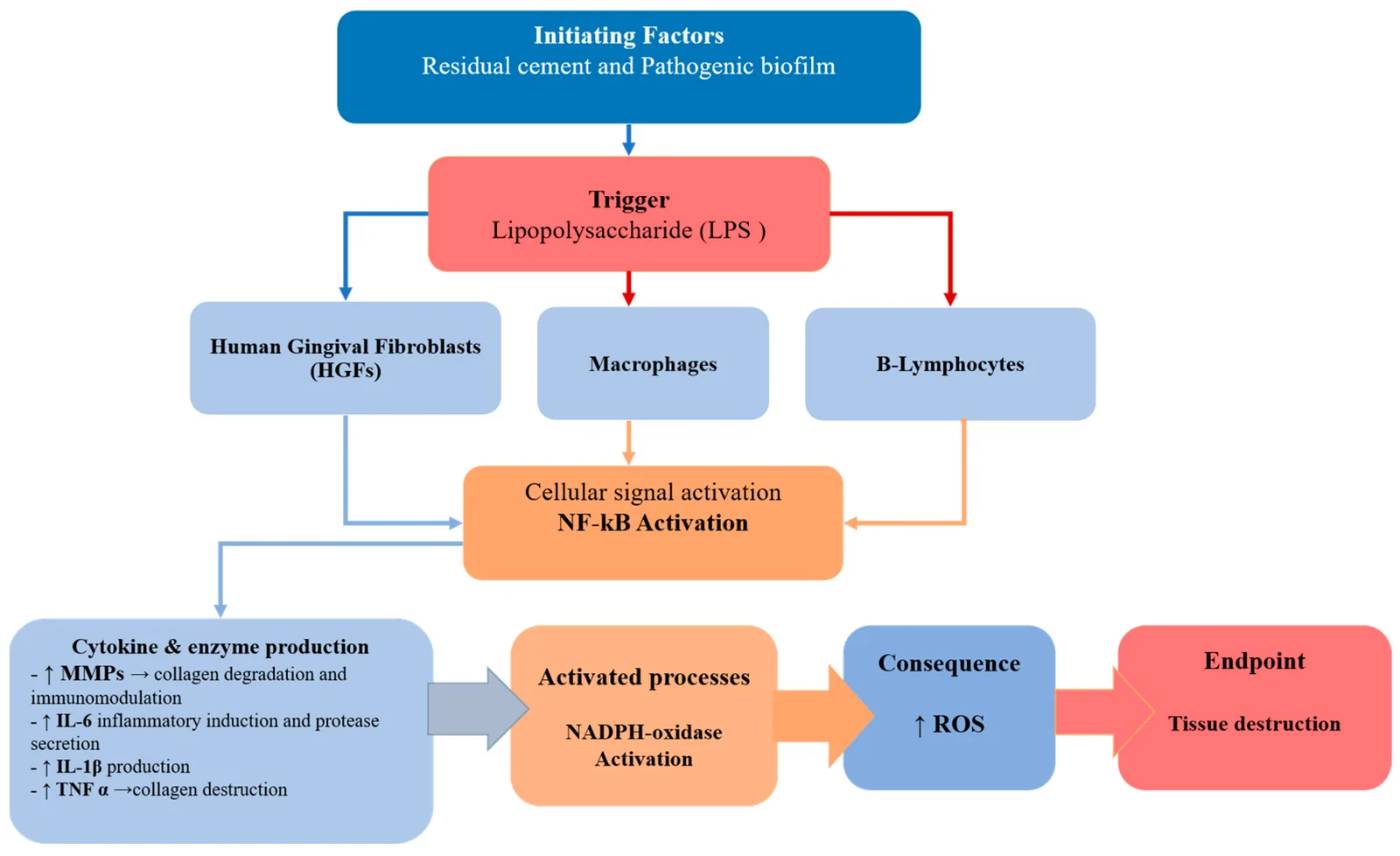
Molecular mechanism of peri-implantitis onset. Note: The upper arrows (↑) denote an increase in the biological parameters. Abbreviations: LPS—lipopolysaccharide, HGF: human gingival fibroblasts; NF-kB—nuclear factor kappa-light-chain-enhancer of activated B cells; MMPs—matrix metalloproteinases; IL 1β/6—interleukin 1β/6; TNF α—tumor necrosis factor-α; ROS—Reactive Oxygen Species.

At the bone interface, inflammatory mediators originating in the overlying soft tissues influence osteoclast–osteoblast coupling. Cement-induced inflammation upregulates the expression of Receptor Activator of Nuclear factor-kappa B Ligand (RANKL) in fibroblasts and immune cells and downregulating Osteoprotegerin (OPG) [25,49]. This profound imbalance between RANKL and OPG levels directly stimulates osteoclast differentiation, driving progressive peri-implant bone resorption. The molecular process by which PG, the primary periodontal pathogen, along with other periodontal irritants (such as **residual cement**) contributes to the onset of peri-implantitis is schematically represented in the figure below (Figure 4).

In conclusion, luting materials influence peri-implant tissue homeostasis through interconnected molecular pathways involving cytotoxic stress, immune dysregulation, impaired epithelial sealing, microbial dysbiosis and osteo-immune imbalance. Residual cement transforms the peri-implant niche from a regulated healing environment into a chronic inflammatory microsystem, providing a mechanistic explanation for cement-associated peri-implant diseases.

A critical comparison of these cytotoxic pathways reveals a profound material-dependent disparity. While traditional luting agents, such as zinc phosphate or glass ionomer cements, primarily exert transient cytotoxicity driven by early pH variations, resin-based cements initiate a more persistent, chronic cellular assault. The continuous, slow release of free methacrylate monomers maintains a state of long-term intracellular oxidative stress, impairing cellular adhesion and signaling pathways long after clinical polymerization. Therefore, evaluating cement cytotoxicity must extend beyond immediate post-setting biocompatibility and critically account for the chronic, low-grade molecular damage that actively compromises the fragile peri-implant mucosal seal.

### 2.5. The Role of the Autophagy in Peri-Implant Inflammation

Autophagy is a highly conserved intracellular degradation pathway involved in regulating the inflammatory response, removing damaged organelles, maintaining cellular homeostasis and balancing energy substrates [55]. Furthermore, it supports cellular adaptation and survival under a variety of stress conditions [56,57]. In recent years, periodontal and peri-implant research has focused heavily on these physiological processes [11], demonstrating that a disruption of autophagy is directly associated with persistence of inflammation and impaired bone regeneration. While direct in vivo evidence demonstrating that residual dental cements modulate autophagy specifically within peri-implant tissues remains limited, this pathological link is strongly inferred from broader molecular pathways governing oxidative stress and chronic inflammation [42].

At the molecular level, the biological complications triggered by residual luting materials are heavily linked to cellular stress. Methacrylate-based resin cements release monomers (such as Bis-GMA, UDMA, TEGMA and HEMA) that interact directly with the surrounding peri-implant soft tissues, inducing severe intracellular imbalance, cell apoptosis and genotoxicity in HGF via overproduction of ROS [20].

Within this microenvironmental stress context, autophagy initially serves as a vital cytoprotective quality-control mechanism. Under moderate or short-term monomer exposure, elevated ROS level acts as upstream signaling molecules that modulate the Protein kinase B (AKT)/mammalian Target of Rapamycin (mTOR) pathway downregulating mTOR complex 1 (mTORC1) to initiate autophagosome formation. This adaptative phase allows cells to capture and degrade damaged mitochondria (mitophagy) and toxic components, thereby maintaining cellular homeostasis and viability [58,59].

However, prolonged and chronic exposure to subgingival residual dental cements breaks this fragile equilibrium and transitions the cytoprotective mechanism into a destructive pathological cascade. Continuous monomer infiltration leads to an excessive, cytotoxic accumulation of ROS that directly disrupts the late-stage fusion between autophagosomes and lysosomes. This blockade of the autophagic flux causes an intracellular buildup of dysfunctional protein aggregates and damaged organelles, marked by a failure to degrade the autophagic cargo receptor Protein 62/Sequestosome 1 (p62/SQSTM1) [23,52].

The link between this autophagic impairment and cement-induced peri-implant bone destruction is established through the hyperactivation of the immune response. The accumulation of undegraded cargo and persistent oxidative stress triggers the assembly and activation of the Nucleotide-binding Oligomerization Domain-like Receptor (NLRP3) inflammasome, which drives the massive extracellular release of pro-inflammatory cytokines, specifically IL-1β and IL-6, alongside TNF-α [48,50].

At the bone interface, these cement-driven pro-inflammatory cytokines diffuse toward peri-implant osteoblastic lines and resident immune cells, heavily upregulating the expression of RANKL while concomitantly downregulating OPG. The resulting high RANKL/OPG ratio accelerates local osteoclastogenesis and differentiation, tipping the homeostatic balance toward aggressive, progressive alveolar bone resorption around the implant. Thus, the chronic failure of the autophagic pathway under residual cement toxicity does not merely represent a loss of cellular viability, but may function as a key molecular bridge that accelerates downstream inflammatory osteolysis, providing a comprehensive mechanistic framework for the clinical tissue destruction observed in late-stage peri-implantitis [25,49].

Nevertheless, it must be explicitly stated that direct in vivo evidence demonstrating that residual dental cements modulate autophagy specifically within peri-implant tissues is currently lacking; instead, this pathological link is indirectly inferred from broader oxidative stress and inflammatory pathways. Consequently, although this concept offers an interesting theoretical model, it remains in its translational infancy and requires robust future validation through specific in vivo research. Until experimental data directly confirms this material-specific autophagic modulation, clinical protocols must avoid overstating translational implications and continue to prioritize mechanical and chemical strategies to minimize residual cement retention.

## 3. Clinical and Translational Implications

From a molecular standpoint, luting materials should be viewed not as passive fixation agents but as active biological modifiers of the peri-implant microenvironment. Minimizing cement excess, selecting biocompatible formulations and promoting retrievable or extraoral cementation techniques are critical strategies to preserve epithelial integrity, immune balance and bone stability [19,25]. Furthermore, clinical success requires careful selection of the prosthetic restoration’s emergence profile, alongside the use of adjunctive paraclinical methods for detecting and visualizing excess cement during routine peri-implant monitoring.

A recent meta-analysis by Atieh et al. [4] concluded that screw retention does not entirely eliminate the risk of peri-implantitis but can significantly reduce it compared to cement-retained restorations; nonetheless, residual cement remains the primary clinical concern. Similarly, research investigating the effects of different dental cements on HGF concluded that no luting agent is biologically inert toward peri-implant tissues [19]. The main risk stems from residual cement, subgingival margin placement and the inherent inability to fully control excess material within the sulcus.

Consequently, clinicians increasingly prefer screw-retained restorations or high controlled cementation techniques such as establishing supragingival margins and applying minimal amount of cement. These recommendations align with practical guidance from an FDI World Dental Federation [12] working group analyzing local risk factors for the onset and progression of peri-implant. Additional preventive measures include designing prosthetic profiles to facilitate oral hygiene, maintaining an adequate zone of keratinized mucosa, treating periodontal and endodontic lesions prior to implant placement and systematically monitoring or reducing deep peri-implant pockets.

### 3.1. Retention Selection: Screw-Retained vs. Cement Retained

Systematic reviews [13,60] report both cemented and screw-retained implant restorations are effective and predictable, provided that cases are individually selected based on mechanical and biological factors. However, longitudinal data reveal distinct complication pattern behaviors. A randomized clinical study [61] of 44 patients receiving implant restorations in esthetic zones found that both retention types exhibited relatively low survival rates over a 5-year period; biological complications were the primary cause of failure for cemented crowns, whereas technical complications predominated for screw-retained restorations. Similar outcomes were documented in another 5-year study involving 34 patients with restorations supported by individualized zirconia abutments [62].

Over a longer follow-up period (7.5 years), clinical evidence indicates that total complication rates increased significantly in favor of cemented restorations [63]. Conversely, a split-mouth clinical study [36] restoring mandibular first molar with one screw-retained on one side and a cemented crown on the contralateral side demonstrated comparable clinical results after 6 months in 14 patients, showing no clear superiority for either retention method within short observation windows. Interestingly, a regional survey revealed a distinct trend toward prioritizing screw-retained restorations among clinicians within their first ten years of practice [2], minimizing the baseline biological risk of cement-induced pathology.

### 3.2. Margin Position and Prosthetic Profile Optimization

Regarding the geometric configuration of the prosthesis, an experimental study [25] concluded that extrusion and persistence of submucosal cement is a major risk factor for late peri-implantitis, as it triggers chronic inflammatory reactions and foreign body-type responses. To facilitate the detection and manual removal of excess cement, studies suggest that crown margins should be positioned supragingivally or no more than 2 mm apical to the gingival crest [3].

Park et al. [64] demonstrated that a wide abutment neck taper geometrically leads to a significantly increased amount of subgingival remnant cement, recommending an emergence profile that is as straight as possible and perfectly aligned with the neck taper of the abutment. Conversely, a concave emergence profile and deep submucosal positioning of restoration margins significantly increase the risk of cement retention [65].

To control internal cement volume and minimize marginal escape, structural modifications such as vent holes are highly effective. While large vents can reduce the adhesive contact surface and compromise mechanical retention [66] recent evidence recommends 1 mm vent holes on the occlusal or lingual surface of implant crowns as the optimal dimension to control excess cement while maintaining adequate retention, thereby balancing esthetic and mechanical requirement [67]. Alternatively, incorporating a properly controlled lingual slot on implant abutments optimizes the retrievability of restorations secured with definite cements without compromising clinical retention [68].

### 3.3. Isolation, Cement Selection and Subgingival Removal Protocols

Ji et al. [69] proposed using minimal cement quantity, reduced seating speed and force and selecting a luting agent with low fluidity. Isolation techniques prove paramount in ensuring effective cement removal. Kuscu et al. [70] concluded that rubber dam is the most effective method for reducing cement residues, particularly for temporary cement, whereas polytetrafluoroethylene (PTFE) tape is ideal when working with resin cement. Conversely, relying solely on a dental probe produced the largest quantity of remnant cement across all tested materials [27].

Cement selection must balance mechanical requirements with biological risk. D. Flanagan [28] concluded that although zinc phosphate cement is radiopaque and its excess can be removed easily, it should be avoided in multiple implant restorations or complex fixed partial denture due to its high solubility and poor mechanical properties, citing modern alternatives like RMGIC as more stable options. To mitigate long-term biological risks, using semipermanent cements for securing zirconia crowns offers the possibility of non-destructive crown removal to facilitate hygiene and monitoring [27].

When subgingival excess does occur, the choice of clearing instruments determines marginal integrity. An in vitro study [71] demonstrated that using Polyether Ether Ketone (PEEK) ultrasonic tips was significantly more effective at removing cement excess around implant-supported restorations than standard manual instrumentation. Meanwhile, resin cement left the least amount of residue regardless of cleaning method; PEEK tips were especially beneficial for GIC and polycarboxylate cements, which otherwise left substantial subgingival residues [69].

### 3.4. Paraclinical Detection and Long-Term Monitoring

A significant clinical challenge is that standard periapical radiographs frequently fail to detect all subgingival residues, especially on non-mesial or non-distal surfaces [65]. Consequently, the radiopacity of the luting agent is crucial. Cal et al. [72] analyzed the radiopacity of various luting cements, concluding that high radiopaque cements are strictly recommended in areas with limited clinical access. Cements containing zinc oxide, barium sulfate, or ytterbium trifluoride (e.g., Poly-F Plus, Fuji Temp LT, and Multilink Implant) are highly visible, whereas materials with radiopacity similar to or less than of dentin (e.g., Implantlink Semi, Premier, and Dentotemp) must be used with caution due to the risk of remaining radiographically invisible.

To bypass the limits of conventional radiography, advanced visualization techniques are required. For instance, optical coherence tomography has proven effective for evaluating and visualizing remnant cements, provided the mucosa thickness is less than 3 mm [73]. Direct visualization via dental endoscopy has also been successfully used to identify the exact location of subgingival residues. A clinical study by Montevecchi et al. [37] identified cement residues most frequently in the maxillary arch of patients who neglected periodic professional maintenance, noting that depth of subgingival cement detection directly correlated with the severity of the subsequent peri-implant disease. This underscores the necessity of strict, systematic recall intervals incorporating visual inspection under magnification, bleeding on probing (BOP) tracking and targeted radiographic or endoscopic monitoring to prevent irreversible bone loss.

The analyzed literature consistently establishes a strong association between the use of dental cements in implant prosthetic and the occurrence of biological complications. Table 2 presents a summary of key studies regarding the types of luting cements used in implant dentistry and clinical strategies employed to mitigate the adverse effects associated with cement excess [19,20,21,22,25,28,42,64,65,67,68,69,70,72,73].

The heterogeneous data summarized in Table 2 emphasize that while conventional isolation and cleaning methods remain critical, they are often insufficient to entirely eliminate subgingival cement remnants. Consequently, this clear clinical limitation highlights the need to look beyond mechanical approaches and explore innovative biomaterial formulations. Transitioning from traditional clinical mitigation strategies to material science optimizations, nanotechnology offers a promising frontier by fundamentally altering the chemical and biological behavior of luting agents. To synthesize these translational findings into concrete chairside workflows, a clinical decision-making matrix is provided in Table 3 [3,8,23,37,67,70,71,72,73].

## 4. Nanotechnology and Advanced Nanoparticles

The development of nanotechnology has opened new perspectives for optimizing dental biomaterials [74]. When critically evaluating the biological impact of luting cements, a clear discrepancy arises between material-induced cytotoxicity and emerging therapeutic solutions. While methacrylate-based remnants directly drive human gingival fibroblasts toward apoptotic pathways via oxidative stress [75], resolving this clinical challenge requires a careful, cross-disciplinary approach.

The clinical relevance of modifying cements with nano-additives extends far beyond mere structural reinforcement. From a translational perspective, structural parameters like film thickness and solubility are directly linked to the pathogenesis of peri-implantitis. A reduced film thickness achieved through nanotechnology ensures tighter marginal adaptation, which mathematically decreases the volume of cement remnants extruded into the peri-implant sulcus during seating. Furthermore, optimizing the dissolution rate prevents early material degradation, minimizing the micro-gap that typically act as niches for anaerobic pathogen colonization [76]. Thus, nano-engineering must be viewed as a dual-action strategy as follows: optimizing mechanical behavior to directly mitigate the primary risk factor of clinical cement retention [77].

Additionally, nano-hydroxyapatite particles (nHAps) have been widely integrated into dental materials to enhance their bioactivity and mechanical properties. While nHAp is widely documented as an implant coating to promote osseointegration, its application as an additive in dental luting cements serves a distinct clinical applicability. When incorporated into glass ionomer or resin-modified glass ionomer cements, nHAp fills structural micro-voids, shifting the homeostatic balance toward active biomineralization at the bone-cement interface. This directly mitigates the localized osteolysis and RANKL upregulation triggered by residual chemical irritants [78,79].

To overcome the cytotoxic and infectious challenges posed by the cement remnants, advanced carbon-based nanomaterials offer a highly translational approach in modern implant dentistry. In particular, graphene nanoparticles (GNPs) and graphene oxide (GO) derivates have stood out due to their exceptional ability to reduce biofilm formation and influence cell signaling [80,81,82,83,84,85]. The integration of GO specifically into dental luting cements is promising due to its dual impact on mechanical behavior and peri-implant tissue response. At concentration ≤ 1 wt.%, GO sheets reinforce the cement matrix, preventing mechanical microfractures under occlusal loading. Biologically, instead of triggering the chronic NLRP3 inflammasome activation seen with traditional resin monomers, preclinical evidence suggests that GO-modified luting agents demonstrate a favorable peri-implant tissue response, potentially protecting HGFs from oxidative stress and maintaining epithelial barrier attachment in experimental models [86,87]. Within the specific framework of implant prosthodontics, these nanostructures successfully counteract risk factors and mitigate the development of peri-implantitis by optimizing surface biocompatibility, accelerating osteointegration and promoting enhanced cell adhesion [76].

Mechanistically, as synthesized in the recent literature [77], graphene oxide architectures exert significant antibiofilm and antibacterial effects by directly disrupting microbial membranes, including localized oxidative stress and interfering with bacterial metabolism [86,88].

This versatility is further demonstrated across various dental applications and hybrid formulation. For instance, incorporating approximately 1 wt.% of graphene oxide into composite resins, polymethyl methacrylate (PMMA) and dental cements have been shown to increase antibacterial resistance by up to 50% [84], while the addition of specialized variants like fluorographene to GIC simultaneously improves mechanical, tribological and antibacterial properties [79].

Graphene-base nanomaterials have shown remarkable properties for tissue engineering in experimental dental models; data indicate that when applied near the implant abutment interface, these nanostructures have the potential to improve the biological response of the surrounding tissues, counteract the destructive inflammatory cascade of peri-implantitis and promote soft and hard tissue regeneration [77].

Recent nano-structural evidence indicates that graphene-based architectures can actively modulate these precise intracellular processes, shifting the balance between autophagy and apoptosis [56]. Graphene nanostructures can fluidly activate or inhibit autophagy by altering ROS levels and modulating the Adenosine monophosphate-activated protein kinase (AMPK)/(mTOR) Unc-51 Like Kinase 1 (ULK1) pathways [55]. This molecular interplay highlights the feasibility of developing advanced bio-intelligent luting cements capable of actively controlling the host anti-inflammatory response through autophagic modulation.

Silver nanoparticles (Ag NPs) have also attracted significant interest in dental research due to their broad-spectrum antimicrobial activity. Magalhaes Moreira et al. evaluated the incorporation of Ag NPs into experimental adhesive cements, demonstrating a significant reduction in biofilm formation without compromising the mechanical properties of the material [89]. In vitro evidence suggests that, when applied to implant prosthodontics models, Ag NPs incorporated into resin-based luting cements can potentially target the dysbiotic multi-species biofilm (such as PG) trapped in subgingival micro-retentive niches. However, their clinical applicability is strictly delimited by a narrow threshold of cytotoxicity. While high concentrations eliminate pathogenic biofilm, they induce mitochondrial failure and autophagic flux blockade in HGFs; therefore, a strict concentration limit of ≤1 wt.% is required to leverage the bacterial potential without compromising the vitality of neighboring peri-implant soft tissue. Nonetheless, a current limitation in this field is that most findings derive from short-term in vitro protocols, highlighting the critical need for standardized in vivo long-term studies.

**Figure 5:**
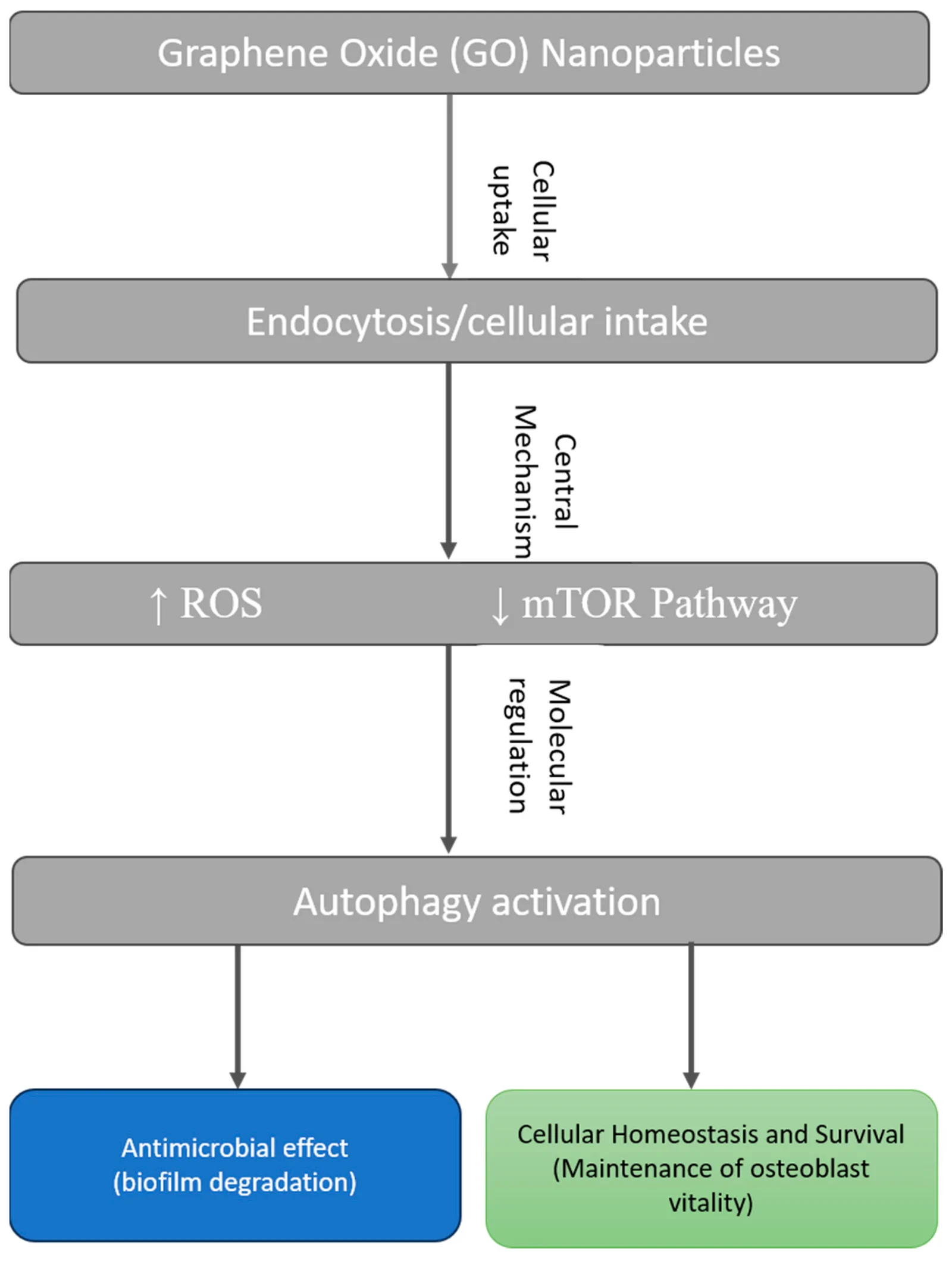
The regulation of autophagy by graphene oxide nanoparticles [55]. Note: ↑—indicates upregulation; ↓—indicates downregulation; GO—graphene oxide; ROS—Reactive Oxygen Species; mTOR—mammalian Target of Rapamycin.

The structural configuration of these nanomaterials dictates their precise intracellular interactions. Unlike Ag NPs, which primarily induce autophagy as a secondary, generic stress response resulting from severe ROS generation and silver ion (Ag^+^) toxicity, GO offers a more precise, tunable and structurally driven regulation [89]. As highlighted by Taheriazam et al., the two-dimensional carbon lattice and rich surface functionalization of GO allow it to interact directly with autophagic machinery and lysosomes, enabling a predictable modulation of autophagic flux compared to less controllable, stress-induced pathways of alternative metal-based nanomaterials [55] (Figure 5). Within the specific framework of implant prosthodontics, hybridization approaches, such as the bio-nanoparticles combining silver and graphene oxide evaluated by Nasim et al. (2020), leverage synergistic pathways to achieve profound antimicrobial efficacy against peri-implant pathogens coupled with low baseline cytotoxicity [76]. This hybrid approach effectively overcomes the biological limitations associated with the standalone incorporation of Ag NPs into resin luting cements, offering a safer more predictable clinical applicability [89].

In addition to nanomaterials, incorporating specific functional organic additives represents another major advancement in enhancing the performance of dental cements. For instance, incorporating 1 wt.% N-acetylcysteine into methacrylate-based resin cements reduced ROS production, prevented DNA double-strand breaks and shielded HGFs from early cellular apoptosis and cell cycle arrest [75]. Similarly, the incorporation of carboxyl-containing additives, such as carboxyl derivatives of phosphazenes, has shown great potential in concurrently addressing multiple clinical demands. These functional groups significantly improve the material’s chemical adhesion to dental hard tissues via structural interactions with hydroxyapatite calcium ions, while inherently promoting antimicrobial activity and biofilm resistance [90].

Importantly, evidence regarding nanotechnology in dentistry cannot be evaluated interchangeably. Data derived from nanostructured dental adhesives, restorative composites or bioactive implant coating do not reflect the specific clinical behavior of luting cements. Luting agents operated under low-viscosity requirements, constant contact with subgingival moisture and unique shear stress dynamics at the titanium-prosthetic interface. Thus, the translational implications of nano-reinforcement discussed here are strictly confined to cement formulation and their direct interaction with the peri-implant microsystem.

Taken together, while these experimental modifications show immense potential, nano-modified luting cements require extensive future investigation, particularly regarding long-term cytotoxicity and dynamic antibacterial behavior to transition safely from in vitro research into standardized clinical practice.

To provide a structured overview of the current state of knowledge, the key findings regarding cement-induced peri-implantitis are categorized into established evidence, emerging evidence and future directions in Table 4.

### Limitations and Future Perspectives

Several limitations must be acknowledged in the current review. First, this study is a narrative review design rather than a systematic methodology, which introduces a potential risk of selection bias and means that a formal quality assessment or risk-of-bias evaluation of the included literature was not performed. Second, much of the discussed data regarding cellular mechanisms, such as macrophage polarization, oxidative stress and biocompatibility, stem predominantly from in vitro and animal model, which may not fully replicate the complex, multi-species biofilm environment of the human peri-implant sulcus. Third, the profound heterogeneity in chemical compositions, radiopacity and mechanical properties among the evaluated dental cements complicates direct clinical comparisons. Finally, while nanotechnology and the modulation of autophagy represent highly promising therapeutic frontiers, there is currently limited direct clinical evidence and a lack of human randomized controlled trials (RCTs) validating their safety, long-term efficacy and clinical translation in treating cement-induced peri-implantitis.

## 5. Conclusions

Dental cements are an essential component of implant prosthetics; however, excess cement is a critical factor in the development of peri-implantitis. Immediate clinical recommendations for mitigating peri-implantitis risk must remain grounded in rigorous, established protocols. Clinicians should focus on minimizing the extrusion of luting agents through precise material selection, prioritizing more biocompatible or easily detectable traditional options over highly cytotoxic resin variants when subgingival margins are present and incorporating strict excess-removal techniques, such as the use of customized abutments or specialized retraction cords. Routine radiographic evaluation and meticulous visual assessment using magnification remain the gold standards for detecting silent cement remnants before irreversible peri-implant tissue destruction occurs.

In contrast, emerging technological avenues offer promising yet distinct future directions. While the molecular pathways linking cement toxicity, ROS production and autophagic dysfunction are well-mapped in laboratory settings, a significant translational gap remains. It is critical to emphasize that current evidence regarding advanced nano-additives, particularly graphene-based architectures, remains strictly preclinical and robust, long-term clinical validation is currently lacking. The challenge for future research lies in transitioning from these speculative in vitro cellular models to standardized, long-term in vivo clinical trials. Only by validating these smart, nano-modified and autophagy-regulating materials in real-world clinical environments can dentistry transition from passively managing the complications of cement remnants to actively preventing peri-implant disease at molecular level.

## Figures and Tables

**Table 1:** The main types of implant restoration luting cement and their properties.

Cement Type	Retention	Solubility	Biocompatibility	Advantages	Disadvantages	Typical Use
Zinc oxide eugenol (ZOE)	Very low	High	Good (bacterial effect)	Easy removal, radiopaque, soothing effect	Low retention, possible marginal gap	Temporary cementation
Non-eugenol zinc oxide (ZOE NE)	Low	Moderate to high	Good (hypoallergenic)	Does not interfere with resin polymerization	Higher microleakage, risk of de-cementation	Temporary cementation
Zinc phosphate (ZP)	Moderate high	Moderate	Acceptable	Good retention, excellent radiopacity, easy excess removal	No chemical adhesion to titanium, poor marginal seal	Semi-permanent cementation
Glass ionomer cement (GIC)	Moderate	Moderate	Good	Fluoride release, chemical adhesion	Moisture sensitive, dimensional instability, not for high-load areas	Semi-permanent/permanent (limited cases) cementation
Resin-modified glass ionomer (RMGIC)	High	Low to moderate	Good	Good retention, radiopaque, improved properties vs. GIC	Water absorption, difficult excess removal	Permanent cementation
Zinc polycarboxylate (ZPC)	Moderate	Moderate	Moderate	Chemical adhesion to tooth structure	Microleakage, possible corrosion with titanium	Permanent, limited use in implant cementation
Resin cements/self-adhesive resin cement (RC)	Very high	Very low	Variable	High retention, insoluble, strong adhesion	Difficult excess removal, high risk of peri-implantitis	Permanent cementation

Note: ZOE: Zinc oxide eugenol cements; ZOE NE: non-eugenol zinc oxide cements; ZP: zinc phosphate cements; GIC: glass ionomer cements; RMGIC: resin-modified glass ionomer cements; ZPC: zinc polycarboxylate cements; RC: resin cements.

**Table 2:** Categorized evidence on luting cement properties, biocompatibility and management strategies.

Category	Authors (Year)	Types of Cements Studied	Key Findings/Relevance
**1. Cement Cytotoxicity and** **Biocompatibility**	P. Bajantri et al. (2022) [19]	GIC (GC Gold Label), ZP (De Trey Zinc), NE ZOE (RelyX Temp NE), RC (RelyX U200)	Maximum cytotoxicity was demonstrated in GIC.
	S. Bajoria et al. (2024) [20]	ZP, GIC, NIB	NIB—the least negative effect on the mineralization.
	F. Diemer et al. (2021) [21]	2 different RC and one conventional ZP	Bone cells show a significantly higher sensitivity and vulnerability to resin-based materials.
	J. Guerrero-Gironésa et al. (2022) [22]	RelyX Unicem 2, Panavia V5, Multilink Hybrid Abutment and SoloCem	Multilink demonstrated the best biocompatibility profile among the tested materials.
	A. Kurt et al. (2018) [42]	RelyX U200, SpeedCEM, G-Cem	Residual monomers from self-adhesive RC induced in vitro cytotoxicity in fibroblast cells.
**2. Remnant Cement Detection**	E. Cal et al. (2017) [72]	Fuji Temp LT, G-Cem LinkAce, Implantlink Semi, Premier, Multilink Implant, Poly-F Plus, Panavia SA, Dentotemp, Fuji Cem 2, Cavex Temporary, RelyX U200, Ketac Cem Plus and Rely X Ultimate	Highly radiopaque cements (such as Poly-F Plus, Multilink Implant, Fuji Temp LT) are appropriate and recommended for luting implant-retained restorations to facilitate clinical detection.
	M. Sanda et al. (2016) [65]	Shofu Hy-Bond Temporary Hard Cement	Optical coherence tomography is effective for evaluating and visualizing remnant cement, provided the mucosa thickness is less than 3 mm.
**3. Cement Removable and Control Strategies**	F. Ji et al. (2024) [69]	Temp Bond NE, Nexus RMGI, MaxCem Elite and RelyX U200	The lowest amount of remnant subgingival cement was observed for RC.
	S. Kuşçu et al. (2025) [70]	ZPC, eugenol-free temporary implant cement and RC	Isolation techniques drastically affect cleanup: the lowest amount of remnant cement after cleaning was observed for RC.
	B. Lakkasetter Chandrashekar et al. (2024) [25]	Bio-ceramic cement	Microtomographic evaluation confirms that remnant cement has a severe negative impact and causes late-stage complications on osseointegrated implants.
**4. Prosthetic Design Factors**	K-H. Choi et al. (2018) [68]	RelyX U200	Incorporating a properly controlled lingual slot on implant abutments optimizes the retrievability of restorations cemented with permanent cements.
	D. Flanagan (2017) [28]	ZP	Due to mechanical and solubility constraints, standard ZP may not be indicated for splints or complex implant-supported fixed partial dentures.
	Y.-H. Park et al. (2022) [64]	RMGIC, ZP, ZOE	A wide abutment neck taper geometrically leads to a significantly increased amount of subgingival remnant cement.
	H. Zhou et al. (2022) [67]	Temp Bond NE	Creating 1-mm mini venting holes on implant crowns is highly recommended to optimally balance cement extrusion control and retention capacity.
**5. Nanomaterial Modification**	L. Sun et al. (2018) [73]	GICs modified with FG	Incorporating FG into GICs has excellent potential to reduce solubility and significantly improve antibacterial properties.

**Table 3:** Clinical decision-making matrix for cement-induced risk mitigation.

Clinical Parameter	Chairside Recommendation and Criteria	Evidence-Based Rationale
**1. Retention Preference**	Select Screw-Retained as first line. Limit Cement-retained to compromised angles or elite esthetic demands.	Eliminates cement foreign-body risk; decreases long-term biological complications compared to cemented crowns.
**2. Margin Position**	Position margins supragingivally or a maximum of 2 mm subgingivally from the gingival crest.	Minimizes undetected cement volume; shallower sulcus depth geometrically facilitates mechanical excess removal.
**3. Structural** **Modification**	Incorporate a 1 mm mini venting hole or a controlled lingual slot on the crown.	Balances internal hydrostatic pressure and controls cement extrusion without sacrificing mechanical retention.
**4. Cement** **Selection Criteria**	Avoid radiolucent options. Select highly radiopaque ZnO or semipermanent formulation. Avoid ZP in complex splints.	Formulations with ZnO, barium sulfate or ytterbium trifluoride provide excellent radiopacity.
**5. Isolation and** **Removal**	Mandate Rubber Dam (temporary cements) or PTFE tape (resin cements). Clear excess using PEEK ultrasonic tips.	Rubber dam/PTFE drastically reduce subgingival flow. PEEK tips clear rigid fragments (GIC/polycarboxylate) without scratching titanium.
**6. Paraclinical Detection**	Combine regular periapical radiographs with dental endoscopy or optical coherence tomograph (mucosa < 3 mm).	Standard X-rays miss non-proximal residues; endoscopy allows direct visualization of deep sulcus remnant.
**7. Follow-up Protocol**	Establish immediate post-cementation baseline X-rays. Schedule 3 to 6-month maintenance recalls tracking BOP and pocket depth.	Early interception of silent cement-mediated foreign body reactions prevent catastrophic late-stage bone loss.

**Table 4:** Synthesis of current evidence on cement-induced peri-implantitis, categorized by level of certainty and future research directions.

Category	Key Focus Areas	Description and Current Scientific Status
Established Evidence	Subgingival cement excess Choice of luting agent Clinical presentation	Undetected subgingival cement excess is a primary cause of peri-implant tissue inflammation and eventual bone loss. Radiotranslucent resin cements pose a significantly higher risk than radiopaque zinc-base cements due to detection difficulty. Clinical retrieval of the implant supported crown or surgical debridement is mandatory to arrest progression.
Emerging Evidence	Cellular mechanisms Biomarkers Autophagy role	Residual cement particles trigger a macrophage-mediated foreign body reaction and severe oxidative stress (ROS accumulation). Local tissue destruction is driven by a localized imbalance in the RANK/OPG ratio and upregulation of MMPs. Dysregulated autophagy pathways (AMPK/mTOR/ULK1) in HGF are linked to cement cytotoxicity.
Future Directions	Nanotechnology Autophagy modulation Clinical translation	Integration of GO or AgNPs into cements to provide long-term antimicrobial properties. Pharmacological targeting of autophagy to enhance cell survival and tissue regeneration around compromised implants. Development of next-generation, smart bioactive cements that minimize biofilm accumulation without losing mechanical properties (requires human RCTs).

## Data Availability

No new data were created or analyzed in this study. Data sharing is not applicable to this article.

## Abbreviations

Ag NPs	Silver nanoparticles
AKT	Protein kinase B
AMPK	Adenosine monophosphate-activated protein kinase
Bis-GMA	Bisphenol A-glycidyl methacrylate
BOP	Bleeding on probing
BPA	Bisphenol A
ECs	Epithelial cells
ECM	Extracellular matrix
GIC	Glass ionomer cement
GNPs	Graphene nanoparticles
GO	Graphene oxide
GSH	Glutathione
HEMA	Hydroxyethyl methacrylate
HGFs	Human gingival fibroblasts
IL-1β/6/10	Interleukin-1β/6/10
LPSs	Lipopolysaccharides
MeSHs	Medical subject headings
MMP	Matrix metalloproteinases
mTOR	Mammalian Target of Rapamycin
NF-kB	Nuclear factor kappa
NIB	Nano-integrated bio-ceramic cement
NLRP3	Nucleotide-binding oligomerization domain-like receptor
NOX	NADPH oxidase
nHAps	Nano-hydroxyapatite particles
OPG	Osteoprotegerin
PEEK	Polyether ether ketone
PG	Porphyromonas gingivalis
PMMA	Polymethyl methacrylate
P62	Protein62
PTFE	Polytetrafluoroethylene
RANKL	Receptor activator of nuclear factor-kappa B ligand
RC	Resin cement
RCTs	Randomized controlled trials
RMGIC	Resin-modified glass ionomer cement
ROS	Reactive oxygen species
SQSTM1	Sequestosome 1
TEGDMA	Triethylene glycol dimethacrylate
TGF-β	Transforming growth factor
TLRs	Toll-like receptors
TNF-α	Tumor necrosis factor-α
UDMA	Urethane dimethacrylate
ULK1	Unc-51 like kinase 1
ZOE	Zinc oxide eugenol
ZOE NE	Non-eugenol zinc oxide
ZP	Zinc phosphate cement
ZPC	Zinc polycarboxylate cement
